# Homeostatic Imbalance of Purine Catabolism in First-Episode Neuroleptic-Naïve Patients with Schizophrenia

**DOI:** 10.1371/journal.pone.0009508

**Published:** 2010-03-03

**Authors:** Jeffrey K. Yao, George G. Dougherty, Ravinder D. Reddy, Matcheri S. Keshavan, Debra M. Montrose, Wayne R. Matson, Joseph McEvoy, Rima Kaddurah-Daouk

**Affiliations:** 1 VA Pittsburgh Healthcare System, Pittsburgh, Pennsylvania, United States of America; 2 Department of Psychiatry, University of Pittsburgh Medical Center, Western Psychiatric Institute & Clinic, Pittsburgh, Pennsylvania, United States of America; 3 Department of Pharmaceutical Sciences, University of Pittsburgh School of Pharmacy, Pittsburgh, Pennsylvania, United States of America; 4 Beth Israel Deaconess Medical Center and Harvard University, Boston, Massachusetts, United States of America; 5 Bedford VA Medical Center, Bedford, Massachusetts, United States of America; 6 Duke University Medical Center, Durham, North Carolina, United States of America; Chiba University Center for Forensic Mental Health, Japan

## Abstract

**Background:**

Purine catabolism may be an unappreciated, but important component of the homeostatic response of mitochondria to oxidant stress. Accumulating evidence suggests a pivotal role of oxidative stress in schizophrenia pathology.

**Methodology/Principal Findings:**

Using high-pressure liquid chromatography coupled with a coulometric multi-electrode array system, we compared 6 purine metabolites simultaneously in plasma between first-episode neuroleptic-naïve patients with schizophrenia (FENNS, n = 25) and healthy controls (HC, n = 30), as well as between FENNS at baseline (BL) and 4 weeks (4w) after antipsychotic treatment. Significantly higher levels of xanthosine (Xant) and lower levels of guanine (G) were seen in both patient groups compared to HC subjects. Moreover, the ratios of G/guanosine (Gr), uric acid (UA)/Gr, and UA/Xant were significantly lower, whereas the ratio of Xant/G was significantly higher in FENNS-BL than in HC. Such changes remained in FENNS-4w with exception that the ratio of UA/Gr was normalized. All 3 groups had significant correlations between G and UA, and Xan and hypoxanthine (Hx). By contrast, correlations of UA with each of Xan and Hx, and the correlation of Xan with Gr were all quite significant for the HC but not for the FENNS. Finally, correlations of Gr with each of UA and G were significant for both HC and FENNS-BL but not for the FENNS-4w.

**Conclusions/Significance:**

During purine catabolism, both conversions of Gr to G and of Xant to Xan are reversible. Decreased ratios of product to precursor suggested a shift favorable to Xant production from Xan, resulting in decreased UA levels in the FENNS. Specifically, the reduced UA/Gr ratio was nearly normalized after 4 weeks of antipsychotic treatment. In addition, there are tightly correlated precursor and product relationships within purine pathways; although some of these correlations persist across disease or medication status, others appear to be lost among FENNS. Taken together, these results suggest that the potential for steady formation of antioxidant UA from purine catabolism is altered early in the course of illness.

## Introduction

Schizophrenia (SZ) is a highly disabling disease characterized by widespread structural and functional brain alterations, the pathogenesis of which remains poorly understood. It has been proposed that at least in part, the neuropathological changes in this illness may result from oxidative stress mechanisms [1]–[10]. The precise components of such alterations remain unclear.

Biological systems have evolved complex protective strategies against free radical toxicity. Under physiological conditions the potential for free radical-mediated damage is kept in check by the antioxidant defense system (AODS), comprising a series of enzymatic and non-enzymatic components. These enzymes act cooperatively at different sites in the free radical pathways. Recently, we have observed that a dynamic state is kept in check during the redox coupling under normal conditions [11]. By contrast, lack of such correlations in brains of SZ patients point to a disturbance of redox coupling mechanisms in the AODS, possibly resulting from a decreased level of glutathione (GSH) as well as age-related decreases of oxidized GSH and glutathione reductase activities. Taken together, our previous data showing altered membrane dynamics and AODS enzyme activities, and findings from other investigators [8]–[10], [12]–[13] are consistent with the notion of free radical-mediated neurotoxicity in schizophrenia [2].

There are multiple pathways to the production of excess free radical generation and subsequent oxidative stress. One such pathway is the formation of peroxynitrite by a reaction of nitric oxide (NO) and superoxide radical. In human brain, NO is metabolized primarily in the form of nitrate. A significantly increased level of NO was found in brains with SZ than those of normal and non-schizophrenic psychiatric controls [14]. Because the reaction of NO with free thiols competes with the same substrate (e.g., GSH), the excessive NO formation may further lead to significant depletion of GSH in SZ.

Purine catabolism may be a previously unappreciated component of the homeostatic response of mitochondria to oxidant stress and may play a critical role in slowing progressive mitochondrial dysfunction in certain disease states [15]. In response to oxidative stress, decreased energy charge or nucleic acid damage, purine metabolism shifts to favor breakdown to xanthine and uric acid.

Novel, powerful and rapid multidimensional separation and characterization methods, e.g., high-pressure liquid chromatography coupled with a 16-channel coulometric multi-electrode array system (HPLC-CMEAS), can lead to revolutionary changes in our understanding at the molecular level [16]–[19]. The resolving power of these methods is superior to one-dimensional approaches, enabling the comprehensive metabolic analyses particularly in the targeted biochemical pathways. These techniques, applied to investigate plasma, can provide valuable insights in the antioxidant defense system involving purine catabolism.

In this study, we compared metabolic signatures consisting of 6 purine-degraded products simultaneously in the plasma between first-episode neuroleptic-naive patients with schizophrenia (FENNS, n = 25) and healthy controls (HC, n = 30) as well as between FENNS at baseline (BL) and 4 weeks (4w) after antipsychotic treatment. Studies of FENNS patients offer an opportunity to investigate neurobiological alterations without the potential confounds of antipsychotic medications and illness chronicity. The data collected from HPLC-CMEAS allow multiple rather than single metabolites to be used in markers for a group, which will greatly improve the predictive value for phenotypes that directly involved in the oxidative stress. More significantly, these comprehensive analyses that generate metabolic profiles represent not only biomarkers for disease but also metabolic maps that can be used to identify specific genes responsible for disease [20]. We hypothesized that the production of antioxidant uric acid would be altered via the purine pathway in the FENNS patients. Such a homeostatic imbalance of purine catabolism may provide us with a biochemical index to predict therapeutic outcome.

## Results

### Tests of Difference in the Group Locations

Descriptive statistics for all the analytes, by group, appear in Table 1. Xanthosine (Xant) had 21 values measured at the same lower threshhold for all groups, i.e., censored below (15 HC values, 2 FENNS-BL, and 4 FENNS-4W), and guanosine (Gr) had 30 values censored below at a lower threshold for all groups (10 HC values, 7 FENNS-BL, and 13 FENNS-4W). These unknown true values thus range from 0 to the respective low threshold values for Xant and Gr. In Tables 1 and 2, these data were used to compute descriptive statistics using their low threshold (tied) values. The medians and quartiles of Table 1 are thus unaffected, while the means and standard deviations will contain systematic errors, as described in the footnotes of each table. The nonparametric Wilcoxon rank-sum and Kendall tau correlation tests use ranks and accommodate tied values. The use of less extreme values in place of the more extreme true values within a small range, may be seen as increasing robustness, whether in numerator or denominator, as for ratios. For the logistic regression classifier, we found that neither Xant nor Gr contributed significantly to the model, whether the threshold values were included or these values were removed.

**Table 1:** Descriptive statistics of metabolites in purine pathway.

Groups	Mean	Median	Firstqrt	Thirdqrt	*p* (HC *vs* FENNS)*	*p* (BL *vs* 4w)†
1. HC						
UA	50606‡	41927	30460	66970		
Xan	183	87	52	171		
Xant	40	11	7.53	22		
G	6.57	6.51	3.07	8.66		
Gr	9.65	7.40	2.82	11		
Hx	1079	354	139	734		
2. FENNS-BL						
UA	37007	25663	20074	46560	0.0193	
Xan	177	70	63	136	0.8210	
Xant	80	34	19	53	**0.0023** §	
G	3.79	1.98	1.12	6.02	0.0156	
Gr	13	7.58	2.82	18	0.5771	
Hx	1190	412	200	865	0.8867	
3. FENNS-4w						
UA	36322	25163	21556	47095	0.0124	0.7510
Xan	103	65	57	98	0.1977	0.1014
Xant	43	25	21	48	**0.0081**	0.5965
G	2.31	1.30	0.84	2.83	**0.0002**	**0.0003**
Gr	7.79	2.82	2.82	8.71	0.2502	0.0405
Hx	662	347	170	817	0.6935	0.4261

* Wilcoxon rank sum test.

† Wilcoxon signed ranks test.

‡ Each value represents ng/mL. Xant and Gr have some left-censored values, so the listed mean estimates may be too large by up to 3.76 (HC), 0.60 (FENNS-BL), 1.20 (FENNS-4W) for Xant, and 0.94 (HC), 0.79 (FENNS-BL), 1.47 (FENNS-4W) for Gr.

§ Significance with p<0.0083 in boldface, trends with p<0.0167, after Bonferroni correction.

**Abbreviations:** HC, healthy control subjects; FENNS, first-episode neuroleptic-naïve patients with schizophrenia; BL, baseline; 4w, 4-week treatment with antipsychotic drugs; UA, uric acid; Xan, xanthine; Xant, xanthosine; G, guanine; Gr, guanosine; Hx, hypoxanthine.

**Table 2:** Comparisons of ratios of product to precursor in purine pathway between healthy control subjects and FENNS patients and between FENNS patients before and after antipsychotic treatment.

Ratios	HC	FENNS-BL	FENNS-4w	*p*		
				HC *vs* BL*	HC *vs* 4w*	BL *vs* 4w†
G/Gr‡	0.89±0.61§	0.37±0.30	0.48±0.72	**0.0004** ¶	**0.0009**	0.8949
Xan/Gr	25.51±42.41	24.88±37.02	24.95±43.27	0.3598	0.5627	0.2872
Xan/G	46.33±85.46	81.92±98.86	66.68±50.91	0.0211	**0.0015**	0.7112
UA/Gr	7371±4325	4152±2193	7047±5556	**0.0015**	0.4967	**0.0025**
UA/G	11998±11525	16529±14751	23771±14948	0.0614	**<0.0001**	0.0236
UA/Xan	508±266	373±246	459±208	0.0393	0.5074	0.0551
Xan/Xant	18.66±31.78	8.54±14.30	7.47±17.33	0.0524	0.0107	0.3388
UA/Xant	5073±4845	1298±972	2184±4310	**0.0021**	0.0067	0.5782
Xan/Hx	0.31±0.18	0.41±0.51	0.31±0.31	0.6689	0.2491	0.2200
UA/Hx	171±124	137±144	145±147	0.1861	0.3255	1.0000
Xant/Gr	7.58±15.35	9.03±13.60	9.40±15.02	0.0892	0.0099	0.2635
Xant/G	10.48±15.58	42.02±75.08	31.35±27.93	**0.0009**	**0.0001**	0.2752
Xant/Hx	0.18±0.36	0.84±2.67	0.20±0.24	0.1805	0.0614	0.7915

* Wilcoxon rank sum test.

† Wilcoxon signed ranks test.

‡ Abbreviations: Gr, guanosine; G, guanine; Xan, xanthine; UA, uric acid; Xant, xanthosine; Hx, hypoxanthine.

§ Mean and standard deviation. Means and standard deviations involving Xant and Gr are somewhat inaccurate due to the use of the low threshhold censoring value in place of the unknown true concentration value.

¶ Significance with p<0.0038 in boldface after the Bonferroni correction.

### Nonparametric Location Testing

The Wilcoxon rank-sum tests showed higher Xant (median, 34.00 ng/mL) in FENNS-BL compared to HC (median, 10.47 ng/mL, *p* = 0.0023, Table 1). After 4-week treatment with antipsychotic treatment, Xant levels (median 24.78 ng/mL) were reduced but remained statistically higher than those of the HC group. Trend-level differences are also noted for uric acid (UA) and guanine (G) between FENNS-BL patients and HC. The values of both UA and G were lower in patient groups than in HC. In addition, G was further reduced in FENNS patients after treatment (p = 0.0003).

The diagnostic groups also showed differences in post-hoc comparisons involving a set of 13 ratios of analytes (Table 2), selected to reflect the product and substrate relationships as described in the purine pathway. The ratios of G/Gr, UA/Gr, and UA/Xant were significantly lower, whereas the ratio of Xant/G was significantly higher in FENNS-BL patients than in HC subjects after the Bonferroni correction, α_corr_ = 0.05/13 = 0.0083. Such differences remained for these patients after 4-week antipsychotic drug treatment with the exception that the ratio of UA/Gr was restored to the levels of the control group. Specifically, the UA/Gr ratio was significantly (p = 0.0025) elevated in FENNS-4w patients as compared with the same individuals at baseline. On the other hand, such a ratio was no longer significantly different between FENNS-4w (7047±5556, mean ± SD) and HC (7371±4325) groups (Table 2).

The effects of covariates gender, age, and Body Mass Index were considered in the analysis. Although there were imbalances of these in the patient and control groups, none of these covariates showed a significant rank correlation with any analyte concentrations for any of the pooled pairs of diagnostic groups. When diagnosis effects upon the analytes were studied *within* covariate-defined subgroupings (male/female, over/under median age, below/above median BMI), significant results were found only for those analytes already showing significance without subgroupings, and these significant differences were in the same directions as the results without the subdividing.

### Logistic Regression Group Classifier

**Figure 1:**
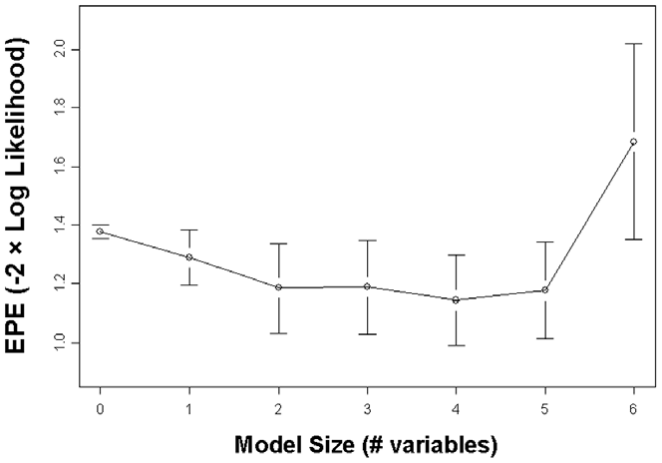
Cross-validation (5-fold) for the logistic regression classifier of HC *vs* FENNS-BL groups. A series of models was chosen by forward selection using as criterion the minimizing of the estimated prediction error (−2 x log likelihood), displayed for each model ± its own standard error. Note that predictors are plasma purine concentrations, only guanine (x = 1) is significant contributor (since its prediction error is within the standard error bar of the 4-variable model, which is the lowest in prediction error). Abbreviations: HC, healthy controls; FENNS-BL, first-episode neuroleptic-naïve schizophrenic patients at baseline.

Furthermore, we built a classification model using logistic regression to assess the performance of the purine pathway analytes in separating the groups in space. In comparing HC to FENNS-BL, lower G (deviance reduction 6.03 on 1 df, Pr(χ^2^) = 0.01) indicated membership in the FENNS-BL group over the HC group. The G model correctly classified 17/25 (68%) FENNS-BL patients and 20/30 (67%) HC subjects. Five-fold cross-validation found that the estimated prediction errors (EPE, approximated by the negative log likelihood loss function) of models with more than one variable were not any better than that of the single-variable G model (Figure 1).

In comparing HC to FENNS-4w, lower G levels (deviance reduction 17.32 on 1 df, Pr(χ^2^) = 0.00003) indicated membership in the FENNS-4w group as opposed to the HC group. The model correctly classified 19/25 (76%) FENNS-4w patients and 21/30 (70%) HC subjects, so neither model achieved highly accurate classification. The cross-validation results found the G model lowest in EPE. None of the analytes made a significant contribution to a model attempting to classify the drug-naive state versus drug-treated state of the patients, whether assessed by deviance reduction or by cross-validation.

### Testing for Nonzero Correlations

**Figure 2:**
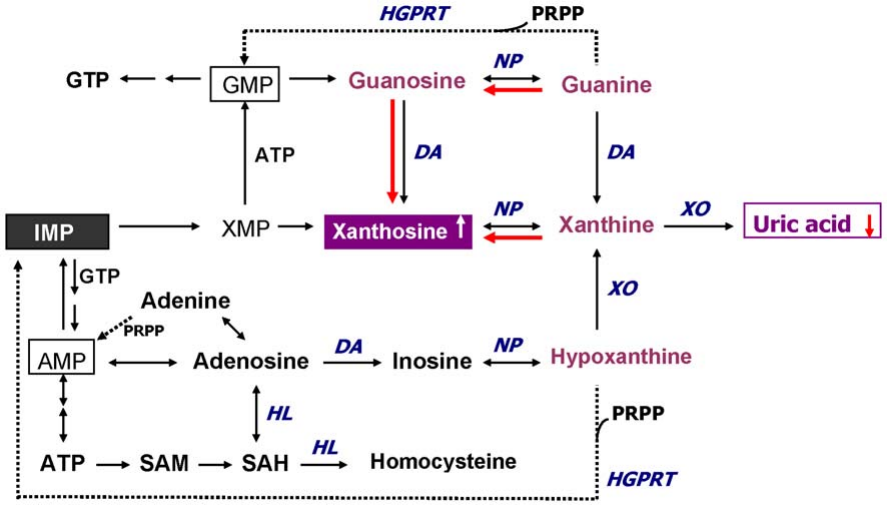
Altered purine catabolism in first-episode neuroleptic-naïve patients with schizophrenia (FENNS). Metabolites identified by purple color were measured in the present study. Red arrows indicate shifts toward an increase of xanthosine and a decrease of uric acid productions in FENNS patients at baseline. Reactions shown with dotted lines represent the “salvage pathways”, which purine bases can be reutilized resulting in considerably energy saving for the cell. Abbreviations: NP, nucleoside phosphorylase; DA, deaminase; HL, hydrolase; XO, xanthine oxidase; HGPRT, hypoxanthine-guanine phosphoribosyl transferase; PRPP, 5-phosphoribosyl pyrophosphate; AMP, adenosine monophosphate; ATP, adenosine triphosphate; SAM, S-adenosylmethionine; SAH, S-adenosylhomocysteine; GMP, guanosine monophosphate; GTP, guanosine triphosphate; IMP, inosine monophosphate; XMP, xanthosine monophosphate.

**Figure 3:**
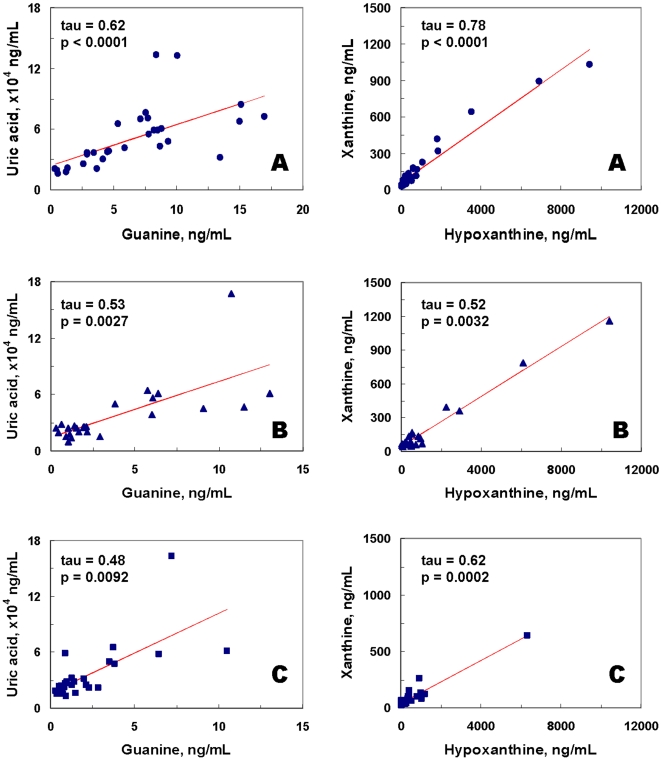
Significant correlations of uric acid to guanine, and xanthine to hypoxanthine in healthy control subjects (A), first-episode neuroleptic-naïve schizophrenic (FENNS) patients at baseline (B), and FENNS patients after 4-week treatment with antipsychotic drugs (C). The *p* values correspond to the computed values of Kendall's tau rank correlations.

We also sought to determine which correlation coefficients among selected pairs of analytes were significantly different from zero (two-tailed). Since the data were not normally distributed, Kendall's Tau rank correlation coefficients were used to test the null hypotheses of τ = 0. Bonferroni confidence intervals, or equivalently, modified *p*-values, were used for control of Type I error. For display purposes, all *p*-values were multiplied by the correction factor of 13, the number of the pairs of purine pathway variables that were of interest to test (Figure 2), i.e., having product-precursor relationships. Thus, *p*-values<0.05 were significant (Figures 3–5); any modified *p*-values >1 were replaced with 1.

**Figure 4 pone-0009508-g004:**
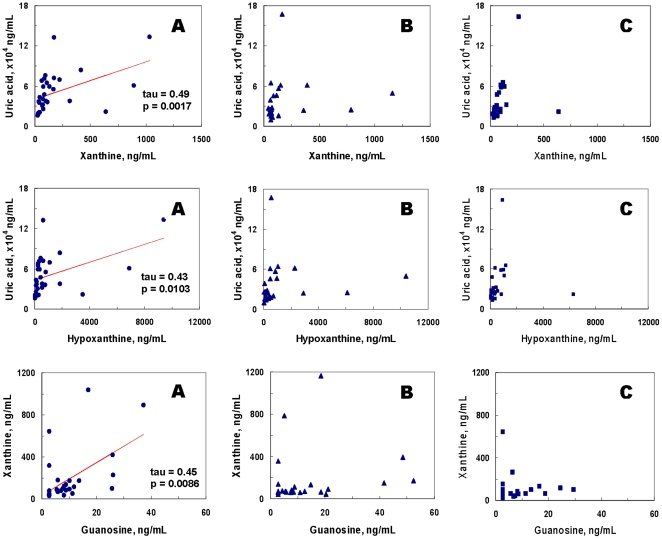
Significant correlations of uric acid to xanthine, uric acid to hypoxanthine, and xanthine to guanosine in healthy control (A) subjects, but not in first-episode neuroleptic-naïve schizophrenic (FENNS) patients at baseline (B) and FENNS patients after 4-week treatment with antipsychotic drugs (C). The *p* values correspond to the computed values of Kendall's tau rank correlations.

**Figure 5:**
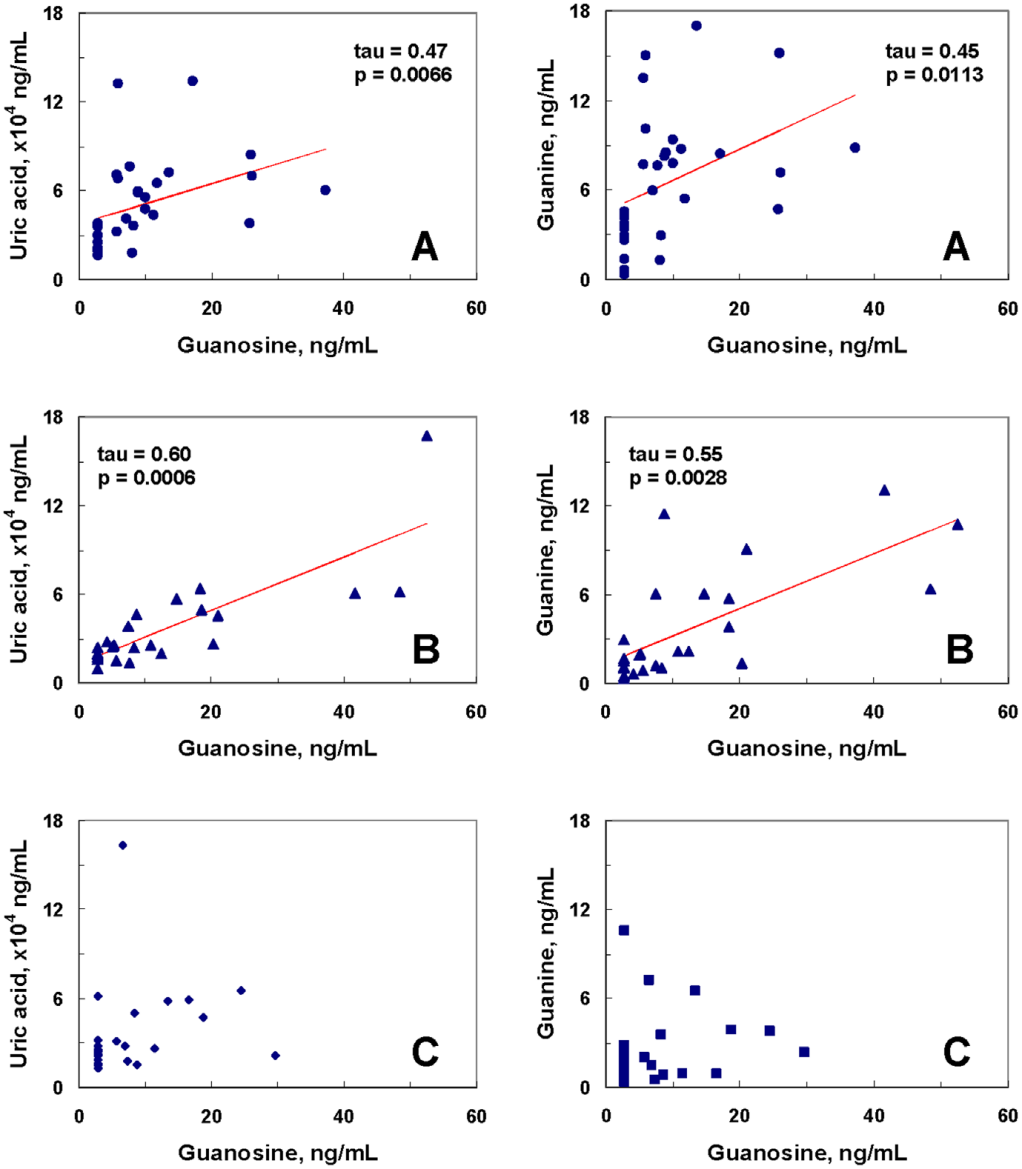
Significant correlations of uric acid to guanosine, and guanine to guanosine in healthy control (A) subjects, and first-episode neuroleptic-naïve schizophrenic (FENNS) patients at baseline (B), but not in FENNS patients after 4-week treatment with antipsychotic drugs (C). The *p* values correspond to the computed values of Kendall's tau rank correlations.

Within the Purines' Pathway, all 3 groups had significant correlations between G and UA, and Xan and Hx (Figure 3). By contrast, correlations of UA with each of Xan and Hx, and correlation of Xan with Gr were all quite significant for the HC group but not for the FENNS group before or after treatment (Figure 4). Finally, correlations of Gr with each of UA and G were significant for both HC and FENNS-BL groups but not for the FENNS group after antipsychotic drug treatment (Figure 5). Care was taken to interpret any disparities in significance of a correlation across the subject groups in concert with other findings, as these disparities were not tested as explicit rejections of hypotheses of zero difference in the correlation coefficients of the respective groups.

## Discussion

### Homeostatic Imbalance of Purine Catabolism

In mammalian cells, purines are synthesized *de novo* from amino acids, formate and carbon dioxide as nitrogen and carbon donors. In addition to their major role in cellular energy metabolism, purines (e.g., ATP) also function as neurotransmitters that are released from neurons or other cells in response to action potentials [21]. Nucleotides (e.g., inosine monophosphate or IMP) are phosphate esters of the purine nucleosides. The tri- and diphosphates of the nucleosides are found in normally functioning cells to a greater extent than the monophosphate nucleosides. IMP is not present in the cells under normal conditions and is converted to adenine, xanthine, and guanine nucleotides by the pathways shown in Figure 2. Under resting conditions, adenosine is derived from S-adenosyl homocysteine (SAH) by the enzyme SAH hydrolase. Extracellular adenosine is rapidly removed in part by conversion to AMP by adenosine kinase, and in part by degradation to inosine by adenosine deaminase. Subsequently, inosine is converted to Hx by the enzyme nucleoside phosphorylase (NP).

During the *de novo* synthesis of purine nucleotides, many reactions require a great deal of energy utilizing the hydrolysis of ATP. To provide “energy saving” for the cell, the purine bases can be reutilized via “salvage pathways” [22] by converting adenine, guanine or Hx to AMP, GMP or IMP, respectively (shown dotted arrow in Figure 2). The unsalvaged Hx is then converted to xanthine (Xan), which is further converted to uric acid (UA) by xanthine oxidase. In man, UA is the final product of purine catabolism [21], which has been implicated as a risk factor and cause of numerous pathological conditions (see below).

In the present study, we evaluated the purine pathway by quantitative determinations of six major purine metabolites consisting of xanthosine (Xant), Xan, Hx, G, Gr, and UA. During the purine degradations, both conversions from Gr to G and from Xant to Xan are readily reversible (Figure 2). Altered ratios of product to precursor, i.e., significantly decreased ratios of G/Gr, UA/Gr and UA/Xant, and the increased ratio of Xant/G (Table 2), suggested a shift favorable to the Xant formation (approximately 2-fold increase) in the FENNS patients. Consequently, the potential for steady formation of antioxidant uric acid from purines was reduced at first testing of the patients, which is shown in the red arrow of Figure 2. More importantly, such an imbalance in purine catabolism is observed independent of treatment since patients were neuroleptic-naïve at entry into the study.

An early study by Kristal et al. [15] suggested that purine catabolism may contribute to mitochondrial antioxidant defense by producing the antioxidant UA. Failure to maintain elevated xanthine and UA occurred contemporaneously with progressive mitochondrial dysfunction. In accordance with our previous findings of significant decreases of plasma UA levels in either FENNS patients [23] or clinically stable patients with chronic SZ [24], the present data provide further support of a defect in the antioxidant defense system in SZ [1]–[10]. Recently, an altered purine catabolism has also been demonstrated in subjects with cocaine addiction [25] or opioid dependence [26], although plasma UA levels remained unchanged. It is not clear whether such changes in purine metabolites without affecting plasma UA levels would eventually lead to oxidative damage in substance abusers.

### Dual Roles of Uric Acid in Antioxidant Defense System (AODS)

Uric acid (UA) is mainly synthesized from adenine- and guanine-based purines by the enzyme xanthine oxidase through the purine catabolism (Figure 2), and seems to be endproduct of purine catabolism in man. The blood levels of UA depend upon the dietary intake of purines, biosynthesis of UA and the rate of UA excretion [27]. Furthermore, the plasma levels of UA are regulated by a renal transport system consisting of glomerular filtration, reabsorption, secretion, and postsecretory reabsorption components [28]. In the majority of mammals (but not in man), UA is further converted to allantoin by urate oxidase (uricase). Contrary to the traditional understanding as a metabolically inert and waste compound without any physiological significance, UA is a major, natural antioxidant contributing to approximately 60% of the free radical scavenging activity in human blood [29].

**Figure 6:**
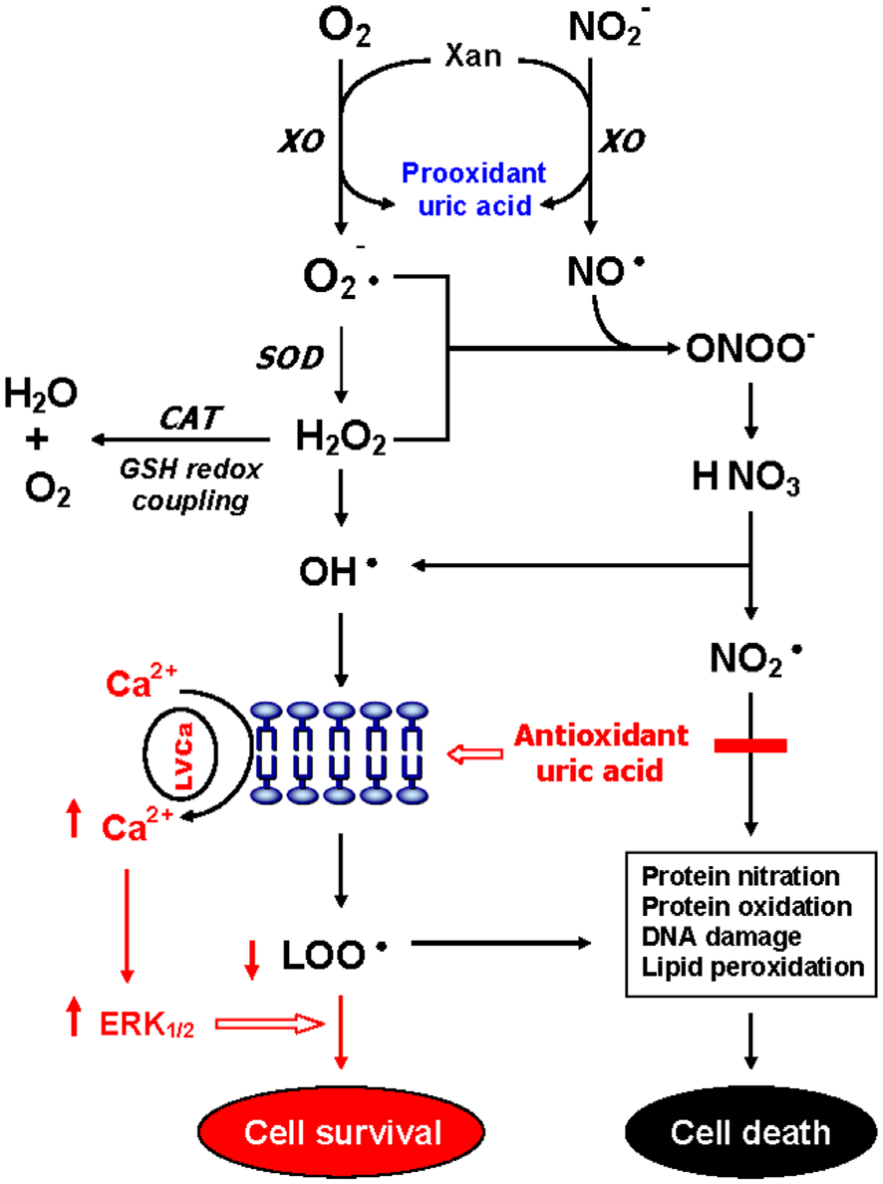
Possible mechanisms involving promotion and removal of free radicals by uric acid. Abbreviations: Xan, xanthine; XO, xanthine oxidase; SOD, superoxide dismutase; CAT, catalase; GSH, glutathione; O_2_^ ^•^, superoxide anion radical; H_2_O_2_, hydrogen peroxide; NO^•^, nitric oxide; ONOO^ ^^, peroxynitrite; NO_2_^•^, nitrogen dioxide radical; OH^•^, hydroxyl radical; LOO^•^, lipid peroxyl radical; LVCa, L-type voltage-gated calcium channel; ERK_1/2_; extracellular signal-regulated kinases_1/2_.

Past studies have demonstrated that UA and inosine (precursor of UA) may be beneficial in the treatment of oxidative stress related neurodegenerative diseases [30]–[34]. Also, UA may assist in the removal of superoxide by preventing the degradation of superoxide dismutase, which subsequently inhibits its reaction with nitric oxide (NO) to form peroxynitrite [35]. UA can also neutralize peroxynitrite [36] and hydroxyl radicals [37] to inhibit protein nitration [38] and lipid peroxidation [39], respectively. Recent investigations further indicated that UA via astroglia may protect dopaminergic neurons from glutamate toxicity [34], [40]. Moreover, UA prevents the propagation of oxidative stress from the extracellular to the intracellular milieu by preserving the integrity of the plasma membrane at the lipid-aqueous interface boundary [41]. High K^+^-induced depolarization amplifies neuroprotection provided by UA through a mechanism involving Ca^2+^ elevation and extracellular signal-regulated kinases½ (ERK_1/2_) activation (Figure 6). Thus, decreased plasma UA levels may reflect decreased ability of the body to prevent superoxide and peroxynitrite from damaging cellular components [27]. At increased levels, however, UA may be considered as a marker of oxidative stress [42], [43] due to accumulation of reactive oxygen species [44]. Therefore, UA can be served as both anti- and prooxidant in the AODS (Figure 6).

Abnormally high levels of plasma (or serum) UA have been related to cardiovascular disease, gout, hypertension, and renal disease [45]–[49], whereas low levels of plasma (or serum) UA have been linked to Alzheimer's disease, multiple sclerosis, optic neuritis, and Parkinson's disease [40], [50]–[54]. Although some studies have indicated that plasma (or serum) UA may play a role in the development or progression of such diseases [45], [47], [49], [55], [56], it remains unclear whether an increased UA contributes to the cause or is simply a consequence of these pathologic conditions [27].

### Lacking Control Mechanism in Purine Catabolism

There are three major purine bases and their corresponding ribonucleosides formed in purine catabolism, consisting of adenine/adenosine, guanine (G)/guanosine (Gr), and hypoxanthine (Hx)/inosine (Figure 2). When comparing the ratios of product and precursor within the purine pathway, Figures 3–4 have shown the existence of tight product-precursor correlations in those conversions of Gr to UA (via G and Xan), and of Hx to UA (via Xan) in the HC subjects. Interestingly, some of these correlations (i.e., conversion of Hx to Xan) persist across disease or medication status, others (i.e., conversions of Gr to Xan, and Xan to UA) are lost among FENNS patients. Although levels of UA are not significantly correlated with Hx in the patient groups (Figure 4), such a lack of correlation is likely due to the insignificant correlation between UA and Xan, and not conversion of Hx to Xan.

Similar findings of lacking a control mechanism used by HC subjects were also demonstrated in the tryptophan pathway from these same FENNS [57], indicating some metabolite interactions within purine catabolism were also altered in FENNS-BL (Figure 2).

### Effect of Antipsychotic Treatment

Using Wilcoxon signed rank test, we found possibly lower levels of plasma UA in FENNS-BL (*p* = 0.0193) and FENNS-4w (*p* = 0.0124) patients than in HC subjects (Bonferroni corrected α_corr_ = 0.10/6 = 0.0167, see Table 1). In the present study, there were no significant differences in FENNS patients (n = 25) before vs after 4 weeks of antipsychotic treatment. Previously, applying the controlled discontinuation of haloperidol in patients with chronic SZ, we have conducted evaluation of antipsychotic drug effects on plasma UA levels [24]. Plasma UA levels were also found to be significantly lower in patients both on- and off-treatment with haloperidol than in normal volunteers. Furthermore, plasma UA levels were marginally significantly higher in patients treated with haloperidol than in the same individuals (n = 34) after haloperidol withdrawal.

On the other hand, there was a significant reduction of guanine (G) in FENNS-4w patients as compared with either HC volunteers or FENNS-BL patients (Table 1). In addition, using the Kendall tau method, significant correlations between Gr and UA (or G) shown in HC and FENNS-BL groups appeared to be lacking in FENNS-4w patients (Figure 5). Our findings indicated that the conversion of Gr to UA through G and Xan intermediates was affected by the antipsychotic drugs, consistent with our data that significant product-precursor correlations between UA and Gr, and between G and Gr were disrupted after 4-w treatment of antipsychotic treatment (Figure 5). Furthermore, the significantly decreased ratio of UA to Gr in FENNS-BL patients could be normalized in FENNS patients after 4w antipsychotic treatment (Table 2); suggesting that UA/Gr ratio may be a biochemical predictor for therapeutic outcome.

Taken together, reduced levels of plasma UA in patients with schizophrenia were not likely the result of antipsychotic treatment. In fact, antipsychotic treatment may increase the concentration of UA through the purine catabolic pathway converting Gr to UA (Figure 2).

### Effect of Other Medications or Pathological Conditions

Reduced levels of UA are seen with a variety of medications (allopurinol, aspirin, probenacid, coumadin, corticosteroids), liver disease, alcohol use, Wilson's disease, hemochromatosis, protein or purine deficiency, xanthine oxidase deficiency, syndrome of inappropriate ADH secretion (SIADH) or renal tubule disease [58]. None of these drugs or pathological conditions are likely to account for low plasma UA in our patients.

In a report of 3 chronic schizophrenic patients, hypouricemia was found in association with long-term polydipsia-hyponatremia syndrome [59]. However, patients in the present study were healthy, with no evidence for liver or kidney disease or significant calorie restriction. The reductions in patients of plasma UA were within normal limits, suggesting that the lowered levels are not indicative of ongoing disease processes (other than SZ) that can affect the antioxidant UA. Rather, the lowered levels may reflect subtle changes indicating either the acute phase response [60], [61] or oxidative stress [2].

### Effect of Cigarette Smoking on Antioxidant Capacity

Patients with schizophrenia smoke at very high rates [62]. Smoking, in turn, is associated with oxidative stress and decreases in antioxidants in the general population [63]–[65]. One of the major compounds in the gas phase of tobacco smoke is nitric oxide. It has been suggested that nitric oxide reacts with smoke olefins to form carbon-centered radicals [63]. On the other hand, the tar phase consists of a semiquinone radical that promotes hydrogen peroxide formation. Moreover, tobacco smoke may increase free radical formation by activating neutrophils.

We have previously demonstrated that plasma antioxidants including uric acid are not affected by smoking status in patients with chronic schizophrenia [24]. Furthermore, there were no significant differences on plasma antioxidant measures between nonsmokers (n = 18) and smokers (n = 13) in the FENNS patients [23]. Among nonsmoking groups, plasma UA levels remained significantly lower in the FENNS patients than in HC subjects [23]. Thus, the altered purine catabolism in schizophrenia cannot be accounted for by smoking alone.

### Effect of Dietary Intake

It is possible that differences between FENNS patients and HC subjects in this study can be accounted for by environmental factors, such as diet, which is known to affect both the antioxidant system and the production of free radicals [66]. All subjects in the present study were recruited from the general population, without a controlled diet. Although specific dietary history was not documented for each subject, the body mass indices (BMI) were higher in normal control subjects than in FENNS (Table 3), suggesting that dietary factors could account for the findings. In a previous study examining plasma UA in patients with chronic SZ who were hospitalized and maintained on a controlled and balanced diet without alcohol consumption [24], a correlation between BMI and plasma UA was found in patients but not normal controls. Moreover, the BMI was higher in patients with chronic SZ than normal controls. By contrast, in the present study the BMI was lower in FENNS patients. Nevertheless, there were significantly lower plasma UA levels present in both neuroleptic-free chronic [24] and treatment-naïve [23] patients with SZ. Thus, it is unlikely that *decreased* plasma uric acid level in SZ patients resulted from alterations in BMI.

**Table 3:** Subject characteristics.

Demographical features	Healthy controls (HC)		FENNS*	
	*Male*	*Female*	*Male*	*Female*
Number	18	12	19	6
Age (yrs)	22.5±4.5	23.2±4.6	21.4±5.5	26.3±10.6
Age of onset (yrs)	---	---	18.8±6.8	23.9±9.54
Illness duration (yrs)	---	---	1.23±1.29	2.43±2.22
Educations (yrs)	14.3±3.1	13.9±2.3	11.8±2.9	12.3±4.6
Weight (kg)	84.3±18.1	63.7±8.8	70.8±19.7†	54.4±8.7‡
Height (cm)	179.3±8.1	164.8±6.6	173.2±10.4	163.3±4.3
Body mass index	26.6±5.4	23.8±3.0	23.9±4.8	20.7±3.0

* FENNS, First-episode neuroleptic-naïve patients with schizophrenia including 10 undifferentiated type, 2 paranoid type, 3 disorganized type, and 2 residual type; 7 schizoaffective disorder; and 1 schizophreniform disorder.

† *p* = 0.0357 (HC-male *vs* FENNS-male, unpaired, 2-tailed *t*-test).

‡ *p* = 0.0597 (HC-female *vs* FENNS-female, unpaired, 2-tailed *t*-test).

### Oxidative Stress and Schizophrenia

Schizophrenia is a major mental disorder without a clearly identified pathophysiology. This is also true for bipolar disorder and major depression. While these conditions have distinctive clinical phenotypes, there appear to be pathophysiological similarities that suggest pathogenetic pathways that may be common to these disorders. There is increasing evidence of antioxidant defense system (AODS) deficits in schizophrenia [1]–[10]. A significant contribution to the body's total antioxidant capacity comes from antioxidant molecules in plasma, such as albumin, uric acid and bilirubin. Significant reductions of plasma antioxidants (e.g., albumin, bilirubin and uric acid) are seen early in the course of schizophrenia [23] and the present study, consistent with previous findings in patients with chronic schizophrenia [2], [7]. More importantly, these reductions are observed independent of treatment since patients were antipsychotic drug-naïve at entry into the study.

The critical antioxidant enzymes including superoxide dismutase, catalase and glutathione peroxidase (Figure 6) act cooperatively at different sites in the free radical pathways. Non-enzymatic antioxidant components, may be equally important in the overall AODS, include uric acid, albumin, bilirubin, glutathione, α-tocopherol (vitamin E), ascorbic acid (vitamin C), and β-carotene. Oxidative stress in brain occurs when the generation of reactive oxygen species overrides the ability of endogenous antioxidant systems to remove excess reactive oxygen species subsequently leading to cellular damage [67]. Increasing evidence suggests that mitochondrial pathology and oxidative stress may be the most critical component in the pathophysiology and outcome of schizophrenia [1]–[10], [68], [69].

As discussed earlier, we [11] have observed that a dynamic state is kept in check during the redox coupling under normal conditions. By contrast, our observation of lack of such correlations in brains with schizophrenia point to a disturbance of redox coupling mechanisms in the antioxidant defense system, possibly resulting from a decreased level of glutathione (GSH) and age-related decreases of oxidized GSH and glutathione reductase activities. In addition, we have also shown a significantly higher level of nitric oxide (NO) in schizophrenia brains than in those of normal and non-schizophrenia psychiatric controls [14]. Taken together, our present data showing homeostatic imbalance of purine catabolism and earlier data showing altered membrane dynamics and antioxidant defense system enzyme activities, and other findings of abnormal GSH gene [6], [9], [70] and SOD gene [71], [72] are consistent with the notion of free radical-mediated neurotoxicity in schizophrenia.

### Limitations

In the present investigation, we have not carried out data analyses of biochemical correlations to clinical measures regarding symptom expression and illness severity because other related pathways and cross-pathway relationships are currently under investigation. To provide a better context, we will seek clustering of, and biological meaning in, finding of group differences. This approach will seek clinical-biochemical associations by targeting key biochemical variables instead of pursuing large-scale multiple testing.

## Materials and Methods

### Subjects

#### First-episode neuroleptic-naïve (FENN) patients

Twenty five patients were recruited (Table 3) in their first episode of psychosis after they provisionally met DSM-IV criteria for schizophrenia, schizophreniform or schizoaffective disorder based on Structured Clinical Interview for DSM Disorders (SCID). The initial diagnostic assessments were performed at consensus diagnostic conferences including SCID and all clinical data, and attended by research faculty and staff, chaired by one of authors (MSK or DM). All subjects signed informed consent after a full explanation of the study. The study was approved by both VA Pittsburgh Healthcare System and the University of Pittsburgh Institutional Review Board. Blood samples were obtained in patients at baseline (FENNS-BL) prior to the initiation of antipsychotic agents. A second set of blood samples were obtained in the same patient individuals about 4 weeks after treatment (FENNS-4w) with one or more of the following antipsychotic drugs: risperidone (n = 17), olanzapine (n = 4), quetiapine (n = 2), aripiprazole (n = 1) and haloperidol (n = 2). The number adds up to more than 25 because of polypharmacy. Clinical symptoms were rated by experienced research clinicians at both time points using standard rating scales: Brief Psychiatric Rating Scale (BPRS) [73], Scale for Assessment of Positive Symptoms (SAPS) [74], Scale for Assessment of Negative Symptoms (SANS) [75], and Global Assessment Scale (GAS) [76]. The clinical symptom scores including BPRS, SAPS and GAS improved from baselines after 4 weeks of treatment with antipsychotic drugs (Table 4).

**Table 4:** Clinical assessments.

Rating scales	Baseline	4-week follow-up	P*
Brief Psychiatric Rating Scale (total)	52.58±8.9	42.91±7.5	0.001
Scale for Assessment of Positive Symptoms	24.29±11.03	14.21±12.14	0.001
Scale for Assessment of Negative Symptoms	45.46±10.35	42.17±8.25	0.072
Global Assessment Scale	30.42±9.3	38.46±11.51	0.001

* Wilcoxon signed ranks test; two-sided.

#### Healthy controls

Subjects (n = 30) recruited from the same neighborhoods were matched to the patients by their age, gender and educational level through local advertisement. Subjects with previous exposure to antipsychotic agents, substance abuse or dependence within the preceding 6 months, systemic medical illness requiring treatment, and neurological disorders were excluded. None of the controls had any personal or family history of psychosis.

### Sample Preparation

All blood samples were collected in the morning after overnight fasting. Samples were prepared for analysis by extraction in acidified acetonitrile and analyzed by liquid chromatography with electrode Coul Array (LCECA) system as previously described [54], [57]. Briefly, freshly drawn blood with anticoagulant citrate dextrose (ACD) was centrifuged at 750×g for 7 minutes to remove RBC and stored at −80°C freezer. 250 µl aliquot of stored sample was mixed with 1 ml of acetonitrile/0.4% acetic acid at −25°C and vortexed for 45–60 sec, then temperature was brought to −15°C in a cold block, and vortexed again for 30–45 sec. Samples were centrifuged for 15 min at 12,000×g at 4°C. In total, 1 ml of the resulting supernate was transferred to a 2 ml screw top vial and evaporated under vacuum. It is critical that the vacuum is sufficient to freeze the sample during this step. The sample was reconstituted in 200 µl of mobile phase A and 100 µl were loaded onto two autosampler vials, one of which was archived at −80°C. Profiles are stable in acetonitrile extract, dried extract and mobile phase diluted extract.

During the sample preparation, pools were created from equal volumes of aliquots of all samples. All assays were run in sequences that include 10 samples, authentic reference standard mixtures of 80 known compounds, pools of all samples and duplicate preparations of the same sample. Duplicates are spaced at short and long intervals through the run to reflect the performance of the total data base. Run orders of all samples in this study were randomized. The sequences minimized possible analytical artifacts during further data processing. Pools and duplicates were used to access the precision of the entire data set. In addition, the pools were used as references for time normalization (stretching). A practical advantage of LCECA for this study is the relative freedom from maintenance events. This is important for the generation of consistent databases from large numbers of samples over extended time periods. In our earlier work we have run LCECA continuously for 24 h per day over 6 months.

### High-Pressure Liquid Chromatography Coupled with Electrochemical Coulometric Array (LCECA) Detection

The LCECA method used in this work has been described earlier [19], [57], [77]. Briefly the liquid chromatographic method employs an A mobile phase (10.3 g l^−1^ sodium pentane sulfonate, 5 ml l^−1^ glacial acetic acid) and a B mobile phase (methanol/acetonitrile/isopropanol 8/1/1, 8 g l^−1^ lithium acetate, 20 ml l^−1^ glacial acetic acid). A gradient is run from 100% A to 100% B over 120 min. The electrochemical array of 16 series coulometric detectors is set from 0 to 900 mv in equal 60 mv increments from detector 1–16. In this mode a compound passing through a coulometric electrode is oxidized by 100% of the thermodynamically possible amount. This results in a characteristic signature for a compound expressed as a ratio on sequential electrodes. This ratio provides a high degree of qualitative certainty, which can be set for any particular study [77]. The gradient and detector conditions typically provide responses at the 500 pg ml^−1^ level (5 pg on column) for ca 1500–2000 compounds in biological samples. In comparison with Mass Spectrometry (MS) a specific thermodynamically determined response ratio and retention time in an LCECA method does not carry as much qualitative certainty as an accurate mass peak or fragmentation pattern in MS/MS. However, in comparison with MS for the classes of compounds measurable on LCECA the sensitivity of ca 500 pg ml^−1^ is typically one to two orders of magnitude lower than can be achieved with MS. As an example we conducted a study directed at identifying metabolites implied by the presence of multiple responses in an LCECA method following Huntington's disease patients treated with phenyl butyrate [78]. The LCECA method employed 40 µl of plasma. It was necessary to concentrate and fractionate 4 ml of plasma to obtain sufficient material for qualitative identification in a parallel LCECA/LCMS system. The LCECA method with 100% efficient electron transfer also has an inherent quantitative control based on integration of the total coulombs of the peaks [78] and calculation of quantity by Faraday's law. Thus, it is independent of such factors as variations in ionization efficiency as a result, for instance, of column bleed.

Inter-laboratory/inter-method comparisons are a field in and of themselves. Well designed studies are highly expensive and have to take into account standards, preparative methods, sample splitting techniques and so on, as well as the instrumentation and parameters of instrument usage. Initial efforts are frequently discouraging. As an example the initial round of the multi center/method ESCOT study for 8 hydroxy 2′deoxyguanosine measurements initially returned values differing by a factor of 1000 with the higher values resulting from source artifacts in a gas chromatography-mass spectrometry (GCMS) method.

Enzyme-linked immunosorbent assay techniques for this same analyte in urine are typically comparable to electrochemical methods for controls and standards but a factor of 2–5 higher in various disorders, whereas GCMS techniques for this analyte in CSF have been reported as 10,000 times higher compared with electrochemical techniques.

### Data Reduction and Analyses

All chromatograms in the study were background corrected (BC) to eliminate the base line drift inherent in gradient profiles. By controlling analytical conditions, the location of any particular peak in a 16-channel 110 min chromatogram was held within ±(5–30) sec through the study. BC files were then sequentially time normalized against a single pool in the middle of the study sequence. A two-step stretching protocol with a multitude of peaks was used. First, proprietary software (ESA, CEAS 512) was used to align 15–20 major peaks in the chromatogram and interpolate the positions between them. Then an additional 20–25 smaller peaks present in most samples were selected from the derivative file and those were realigned, keeping the major peaks in the same position. Selected peaks were aligned within ±0.5 sec and non-selected peaks within ±(1–1.5) sec over the entire 110 min assay.

Data was exported in three formats. First all responses matching the retention and EC signature of compounds in the reference standard were exported in concentration units of ng ml^−1^. Second all responses matching resolved peaks in the pool of all samples were exported in terms of their relative response to the pool value. The concentrations of these were subsequently estimated by the total coulombs in the peak assuming a molecular weight of 200 and a two-electron charge transfer.

Third, in addition to determining the concentration levels of peaks against standards and pools, we exported the analytical information for all samples in digital format (digital maps) [78]. Using complete digital output served two purposes: (i) capture of all analytical information for the following data analysis and (ii) avoiding possible artifacts introduced by peak-finding algorithms. The number of variables in the digital maps depends on the resolution set during the data export. In this work, the resolution was set at 1.5 s and the number of data points (variables, defined as the signal at a given time on a given channel) obtained from one sample, using our current LCECA approach, was 66 000. It is important to note that the number of variables in digital maps is not equivalent to the number of analytes because an individual analyte is represented by more than one variable. Depending on the concentration of an analyte and on its separation across the electrochemical array, the number of variables characterizing an analyte could be between 10 and 100. In the consolidated files of a study all variables were aligned in a spreadsheet for data analysis with each column representing a single subject (sample) organized by time from channel 1 to 16. Each row in a spreadsheet represents the response of a compound (variable) at a specific time and channel for all samples. This approach avoids artifacts in data reduction and protects against overfitting in the data analysis. Before data analysis, rows in the digital maps for which all values were negative or <30 pA (noise level of the analytical method), for all samples were eliminated. The data were analysed by conventional statistical methods and by partial least squares discriminant analysis [79]. After finding the variables differentiating the groups (for example, schizophrenic *vs* healthy controls), we sorted the variables by retention time and channel. This step allowed isolation of ‘peak clusters’ (that is, all digital map variables characterizing one specific analyte), which, in turn, provides an identification of specific markers. Then the most significant variables in the digital maps were used to identify the location of the actual marker peaks within the chromatograms.

### Analysis of Purine Pathway

Generally, a metabolic pathway consists of sequential biochemical reactions that generate various products from a set of precursors. Thus, connections between biochemical reactions through the substrate and the product metabolites form complex metabolic networks that may be analyzed using network theory, stoichiometric analysis, and information on protein structure/function and metabolite properties [80]. In this study, estimates of various enzyme activities in purine pathways were calculated using the product to precursor ratios for each enzyme. The following ratios were used: G/Gr, Xan/Xant, Xan/Gr, UA/Gr, UA/Xant, and Xant/G, for purine nucleoside phosphorylase; Xan/G and Xant/Gr, UA/G for deaminase; UA/Xan, Xan/Hx, UA/Hx, and Xant/Hx for xanthine oxidase. The absolute value for each purine metabolite was used for calculating the ratios. Significant differences in such ratios were sought between patients, off or on treatment with antipsychotic drugs, and healthy controls.

### Statistical Analyses

**Figure 7:**
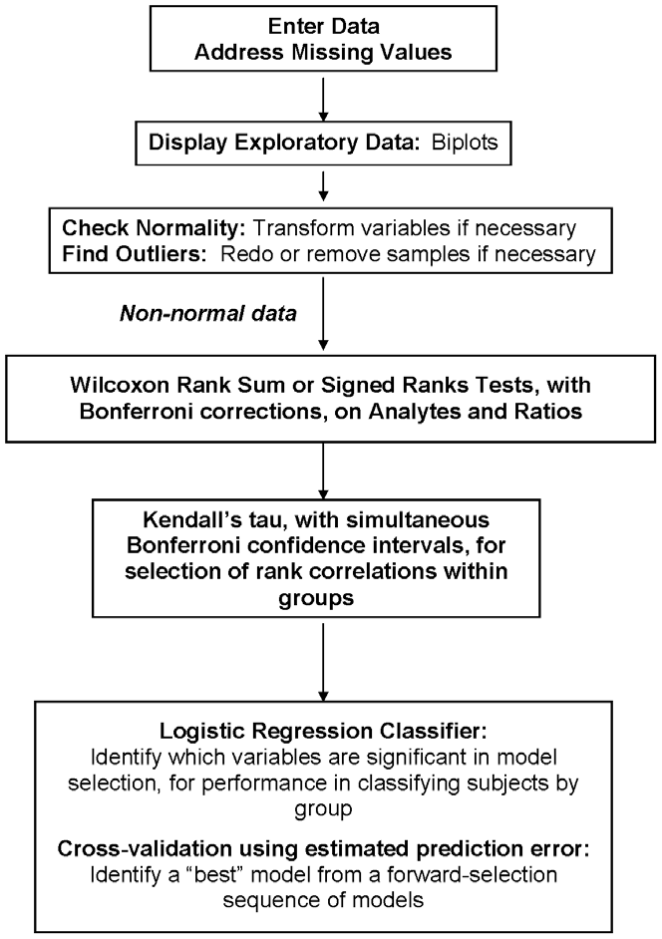
Flow chart of data analyses.

All analyte variables were expressed as ng/ml of plasma. Three groups of samples were analyzed: controls (HC, n = 30), first-episode neuroleptic-naïve schizophrenia patients at baseline (FENNS-BL, n = 25), and the same patients after 4 weeks of antipsychotic treatment (FENNS-4w, n = 25). Six analytes within the purine pathway were measured, and descriptive statistics were computed for each of the 3 groups according to the flow chart for data analyses (Figure 7). The raw data were viewed by quantile-quantile normal and chi-square plots, and by variable-pair scatterplots, to assess normality and nonlinear relationships. Some analytes had a number of identical minimum entries that had been censored below. Not all analytes were normally distributed, so hypotheses of no difference between the relative concentrations of each analyte for pairs of the subject groups (HC, FENNS-BL, FENNS-4W), were tested by nonparametric Wilcoxon rank sum tests or Wilcoxon signed ranks tests, as appropriate. When ties were present, the normal approximations for the above Wilcoxon test statistics [81] were used in the tests. Control of Type I error for this multiple testing process was effected by applying the Bonferroni correction.

We used the rank correlation method of Kendall to examine associations among all 6 analyte variables, within each subject group. This method tests the null hypothesis of no rank correlation among 2 variables whose distribution is not known, and can accommodate tied or censored values up to the extreme case of variables with dichotomous values [82]. Because two correlations, those between Hx and G and between Hx and Gr, did not have product-precursor relationships (Figure 2), we tested the remaining [6×(6−1)/2]−2 = 13 Kendall tau-a correlations against the two-sided alternative, using Bonferroni-corrected α = 0.05/13 = 0.0038 (or α = 0.10/13 = 0.0077), to determine which correlations were clearly (or possibly) different from zero, for each group separately.

Gender, age, and body mass index (BMI) were available for all but two control subjects, but some imbalances were present across diagnostic groups (Table 3). The effects of gender groupings on metabolite variables were examined by Wilcoxon rank sum tests, and the associations of continuous covariates with the metabolites were examined by Kendall's tau correlations, to rule out covariate effects masquerading as diagnostic group differences. We also sought to discover any significant reversals of effects of diagnostic groups across covariates, by assessing diagnosis effects on metabolites within each one of pairs of subgroups defined by 2 levels of the covariates (male/female, below/above median age, below/above median BMI). We sought rejections of the hypotheses of no difference, with α set at the same level (Bonferroni correction only for number of analytes) as for the unsubdivided diagnostic group Wilcoxon testing, α_corr_ of 0.5/6 = 0.0083 (two-tailed), i.e., liberally admitting additional tests per analyte without further correction.

Finally, the ability of the analytes to classify subjects by group membership was examined by logistic regression [83]. A sequence of models was generated by forward selection. Models were assessed by deviance reduction, as well as by 5-fold cross-validation, with estimated prediction error (EPE) approximated by the negative log likelihood loss function [84]. K-fold cross-validation is used to avoid “model error,” i.e., choosing models suggested by random aspects of the data. One divides the N data samples randomly into K nearly-equal portions, and leaves each portion out, successively, while making K estimations of the model parameters, giving K models. Then each of the K models is used to compute the EPE for the corresponding left-out portion of the data. The means and standard errors of the K EPE values are then plotted as the process is repeated for the original large sequence of models (with different numbers of analyte variables). A common procedure for identifying a “best” model is to first find the model with the lowest EPE value (see Figure 1), and then to select the smallest model with EPE within one standard error of the lowest EPE value [84].

## References

[1] Reddy R, Yao JK (1996). Free radical pathology in schizophrenia: A review.. Prostagland Leuko Essen Fatty Acids.

[2] Yao JK, Reddy RD, van Kammen DP (2001). Oxidative damage and schizophrenia: an overview of the evidence and its therapeutic implications.. CNS Drugs.

[3] Mahadik SP, Parikh V, Khan MM, Peet M, Glen I, Horrobin D (2003). The role of oxidative stress in modulating membrane and phospholipid function in schizophrenia.. Phospholipid Spectrum Disorders in Psychiatry and Neurology.

[4] Ben-Shachar D, Laifenfeld D (2004). Mitochondria, synaptic plasticity, and schizophrenia.. Int Rev Neurobiol.

[5] Fendri C, Mechri A, Khiari G, Othman A, Kerkeni A (2006). Oxidative stress involvement in schizophrenia pathophysiology: a review.. Encephale.

[6] Gysin R, Kraftsik R, Sandell J, Bovet P, Chappuis C (2007). Impaired glutathione synthesis in schizophrenia: Convergent genetic and functional evidence.. Proc Natl Acad Sci U S A.

[7] Pillai A, Reddy R, Yao JK, Reddy R, Yao JK (2007). Oxidative stress in schizophrenia.. Fatty acids and oxidative stress in neuropsychiatric disorders.

[8] Othmen LB, Mechri A, Fendri C, Bost M, Chazot G (2008). Altered antioxidant defense system in clinically stable patients with schizophrenia and their unaffected siblings.. Prog Neuropsychopharmacol Biol Psychiatry.

[9] Matsuzawa D, Hashimoto K, Hashimoto T, Shimizu E, Watanabe H (2009). Association study between the genetic polymorphisms of glutathione-related enzymes and schizophrenia in a Japanese population.. Am J Med Genet B Neuropsychiatr Genet.

[10] Virit O, Altindag A, Yumru M, Dalkilic A, Savas HA (2009). A Defect in the Antioxidant Defense System in Schizophrenia.. Neuropsychobiology.

[11] Yao JK, Leonard S, Reddy RD (2006). Altered glutathione redox state in schizophrenia.. Disease Markers.

[12] Ranjekar PK, Hinge A, Hegde MV, Ghate M, Kale A (2003). Decreased antioxidant enzymes and membrane essential polyunsaturated fatty acids in schizophrenic and bipolar mood disorder patients.. Psychiatry Res.

[13] Matsuzawa D, Obata T, Shirayama Y, Nonaka H, Kanazawa Y (2008). Negative correlation between brain glutathione level and negative symptoms in schizophrenia: a 3T ^1^H-MRS study.. PLoS One.

[14] Yao JK, Leonard S, Reddy RD (2004). Increased nitric oxide radicals in postmortem brains from schizophrenic patients.. Schizophr Bull.

[15] Kristal BS, Vigneau-Callahan KE, Moskowitz AJ, Matson WR (1999). Purine Catabolism: Links to Mitochondrial Respiration and Antioxidant Defenses?. Arch Biochem Biophys.

[16] Yao JK, Cheng P (2004). Determination of multiple redox-active compounds by high-performance liquid chromatography with coulometric multi-electrode array system.. J Chromatogr B.

[17] Rozen S, Cudkowicz ME, Bogdanov M, Matson WR, Kristal BS (2005). Metabolomic analysis and signatures in motor neuron disease.. Metabolomics.

[18] Kaddurah-Daouk R, Kristal BS, Weinshilboum RM (2008). Metabolomics: A Global Biochemical Approach to Drug Response and Disease.. Ann Rev Pharmacol Toxicol.

[19] Kristal BS, Shurubor YI, Kaddurah-Daouk R, Matson WR (2007). High-performance liquid chromatography separations coupled with coulometric electrode array detectors: a unique approach to metabolomics.. Methods Mol Biol.

[20] Watkins SM (2000). Comprehensive lipid analysis: a powerful metanomic tool for predictive and diagnostic medicine.. Isr Med Assoc J.

[21] Linden J, Rosin DL, Siegel GJ, Albers RW, Brady ST, Price DL (2006). Purinergic system.. Basic Neurochemistry, Molecular, Cellular and Medical Aspects, 7^th^ edition.

[22] Cory JG, Devlin TM (1982). Purine and pyrimidine nucleotide metabolism.. Text Book of Biochemistry with Clinical Correlations.

[23] Reddy RD, Keshavan MS, Yao JK (2003). Reduced plasma antioxidants in first-episode patients with schizophrenia.. Schizophr Res.

[24] Yao JK, Reddy R, van Kammen DP (1998). Reduced level of plasma antioxidant uric acid in schizophrenia.. Psychiatry Res.

[25] Patkar1 AA, Rozen S, Mannelli P, Matson W, Pae C-U (2009). Alterations in tryptophan and purine metabolism in cocaine addiction: a metabolomic study.. Psychopharmacology.

[26] Mannelli P, Patkar1 A, Rozen S, Matson W, Krishnan R (2009). Opioid use affects antioxidant activity and purine metabolism: preliminary results.. Hum Psychopharmacol.

[27] Kutzing MK, Firestein BL (2007). Altered uric acid levels and disease states.. J Pharm Exp Therapeutics.

[28] Mount DB, Kwon CY, Zandi-Nejad K (2006). Renal urate transport.. Rheum Dis Clin North Am.

[29] Ames BN, Cathcart R, Schwiers E, Hochstein P (1981). Uric acid provides an antioxidant defense in humans against oxidant- and radical-caused aging and cancer: a hypothesis.. Proc Natl Acad Sci U S A.

[30] Hooper DC, Scott GS, Zborek A, Mikheeva T, Kean RB (2000). Uric acid, a peroxynitrite scavenger, inhibits CNS inflammation, blood-CNS barrier permeability changes, and tissue damage in a mouse model of multiple sclerosis.. FASEB J.

[31] Scott GS, Spitsin SV, Kean RB, Mikheeva T, Koprowski H (2002). Therapeutic intervention in experimental allergic encephalomyelitis by administration of uric acid precursors.. Proc Natl Acad Sci U S A.

[32] Spitsin S, Hooper DC, Leist T, Streletz LJ, Mikheeva T (2001). Inactivation of peroxynitrite in multiple sclerosis patients after oral administration of inosine may suggest possible approaches to therapy of the disease.. Mult Scler.

[33] Liu F, You SW, Yao LP, Liu HL, Jiao XY (2006). Secondary degeneration reduced by inosine after spinal cord injury in rats.. Spinal Cord.

[34] Du Y, Chen CP, Tseng CY, Eisenberg Y, Firestein BL (2007). Astroglia-mediated effects of uric acid to protect spinal cord neurons from glutamate toxicity.. Glia.

[35] van der Veen RC, Hinton DR, Incardonna F, Hofman FM (1997). Extensive peroxynitrite activity during progressive stages of central nervous system inflammation.. J Neuroimmunol.

[36] Keller JN, Kindy MS, Holtsberg FW, St Clair DK, Yen HC (1998). Mitochondrial manganese superoxide dismutase prevents neural apoptosis and reduces ischemic brain injury: suppression of peroxynitrite production, lipid peroxidation, and mitochondrial dysfunction.. J Neurosci.

[37] Davies KJ, Sevanian A, Muakkassah-Kelly SF, Hochstein P (1986). Uric acid-iron ion complexes. A new aspect of the antioxidant functions of uric acid.. Biochem J.

[38] Pacher P, Beckman JS, Liaudet L (2007). Nitric oxide and peroxynitrite in health and disease.. Physiol Rev.

[39] Muraoka S, Miura T (2003). Inhibition by uric acid of free radicals that damage biological molecules.. Pharmacol Toxicol.

[40] de Lau LM, Koudstaal PJ, Hofman A, BretelerMM (2005). Serum uric acid levels and the risk of Parkinson disease.. Ann Neurol.

[41] Guerreiro S, Ponceau A, Toulorge D, Martin E, Alvarez-Fischer D (2009). Protection of midbrain dopaminergic neurons by the end-product of purine metabolism uric acid: potentiation by low-level depolarization.. J Neurochem.

[42] Becker BF (1993). Towards the physiological function of uric acid.. J Free Radic Biol Med.

[43] Strazzullo P, Puig JG (2007). Uric acid and oxidative stress: relative impact on cardiovascular risk?. Nutr Metab Cardiovasc Dis.

[44] Hyden MR, Tyagi SC (2004). Uric acid: a new look at an old risk marker for cardiovascular disease, metabolic syndrome, and type 2 diabetes mellitus: the urate redox shuttle.. Nutr Metab (Lond).

[45] Jossa F, Farinaro E, Panico S, Krogh V, Celentano E (1994). Serum uric acid and hypertension: the Olivetti heart study.. J Hum Hypertens.

[46] Freedman DS, Williamson DF, Gunter EW, Byers T (1995). Relation of serum uric acid to mortality and ischemic heart disease. The NHANES I Epidemiologic Follow-up Study.. Am J Epidemiol.

[47] Kang DH, Nakagawa T, Feng L, Watanabe S, Han L (2002). A role for uric acid in the progression of renal disease.. J Am Soc Nephrol.

[48] Choi HK, Mount DB, Reginato AM (2005). Pathogenesis of gout.. Ann Intern Med.

[49] Bos MJ, Koudstaal PJ, Hofman A, Witteman JC, Breteler MM (2006). Uric acid is a risk factor for myocardial infarction and stroke: the Rotterdam study.. Stroke.

[50] Church WH, Ward VL (1994). Uric acid is reduced in the substantia nigra in Parkinson's disease: effect on dopamine oxidation.. Brain Res Bull.

[51] Toncev G, Milicic B, Toncev S, Samardzic G (2002). Serum uric acid levels in multiple sclerosis patients correlate with activity of disease and blood-brain barrier dysfunction.. Eur J Neurol.

[52] Knapp CM, Constantinescu CS, Tan JH, McLean R, Cherryman GR (2004). Serum uric acid levels in optic neuritis.. Mult Scler.

[53] Kim TS, Pae CU, Yoon SJ, Jang WY, Lee NJ (2006). Decreased plasma antioxidants in patients with Alzheimer's disease.. Int J Geriatr Psychiatry.

[54] Bogdanov M, Matson WR, Wang L, Matson T, Saunders-Pullman R (2008). Metabolomic profiling to develop blood biomarkers for Parkinson's disease.. Brain.

[55] Saito I, Saruta T, Kondo K, Nakamura R, Oguro T (1978). Serum uric acid and the renin-angiotensin system in hypertension.. J Am Geriatr Soc.

[56] Waring WS, Webb DJ, Maxwell SR (2000). Uric acid as a risk factor for cardiovascular disease.. Q J Med.

[57] Yao JK, Dougherty GG, Reddy RD, Keshavan MS, Montrose DM (2009). Altered interactions of tryptophan metabolites in first-episode neuroleptic-naïve patients with schizophrenia.. Mol Psychiatry (advance online publication, 28 April 2009).

[58] Fischbach FT (1999). A manual of laboratory and diagnostic tests..

[59] Hanihara T, Amagai I, Hagimoto H, Makimoto Y (1997). Hypouricemia in chronic schizophrenic patients with polydipsia and hyponatremia.. J Clin Psychiatry.

[60] Maes M, Delange J, Ranjan R, Meltzer HY, Desnyder R (1997). Acute phase protein in schizophrenia, mania and major depression: modulation by psychotropic drugs.. Psychiatry Res.

[61] Maes M, Chiavetto LB, Bignotti S, Tura GB, Pioli R (2000). Effects of atypical antipsychotics on the inflammatory response system in schizophrenic patients resistant to treatment with typical neuroleptics.. Eur Neuropsychopharmacol.

[62] Lohr JB, Flynn K (1992). Smoking and schizophrenia.. Schizophr Res.

[63] Pryor WA, Stone K (1992). Oxidants in cigarette smoke.. Ann New York Acad Sci.

[64] Chow KC, Packer L, Fuchs J (1992). Vitamin E and cigarette smoking-induced oxidative damage.. Vitamin E in health and disease.

[65] Stegmayr B, Johansson I, Huhtasaari F, Asplund K (1993). Use of smokeless tobacco and cigarettes–Effects on plasma levels of antioxidant vitamins.. Inter Vitamin Nutr Res.

[66] Papas AM (1996). Determinants of antioxidant status in humans.. Lipids.

[67] Ernster I, Yagi K (1993). Lipid peroxidation in biological membranes: mechanisms and implications.. Active oxygen species, lipid peroxides, and antioxidants.

[68] Whatley SA, Curti D, Das Gupta F, Ferrier IN, Jones S (1998). Superoxide, neuroleptics and the ubiquinone and cytochrome b5 reductases in brain and lymphocytes from normals and schizophrenic patients.. Mol Psychiatry.

[69] Bubber P, Tang J, Haroutunian V, Xu H, Davis KL (2004). Mitochondrial enzymes in schizophrenia.. J Mol Neurosci.

[70] Gysin R, Riederer IM, Cuenod M, Do KQ, Riederer BM (2009). Skin fibroblast model to study an impaired glutathione synthesis: consequences of a genetic polymorphism on the proteome.. Brain Res Bull.

[71] Akyol O, Yanik M, Elyas H, Namli M, Canatan H (2005). Association between Ala-9Val polymorphism of Mn-SOD gene and schizophrenia.. Prog Neuropsychopharmacol Biol Psychiatry.

[72] Walss-Bass C, Soto-Bernardini MC, Johnson-Pais T, Leach RJ, Ontiveros A (2009). Methionine sulfoxide reductase: a novel schizophrenia candidate gene.. Am J Med Genet B Neuropsychiatr Genet.

[73] Overall JE, Gorham DR (1962). The Brief Psychiatric Rating Scale.. Psychol Rep.

[74] Andreasen NC (1984). Scale for the Assessment of Positive Symptoms..

[75] Andreasen NC (1983). Scale for the Assessment of Negative Symptoms..

[76] Endicott J, Spitzer RL, Fleiss JL, Cohen J (1976). The Global Assessment Scale: a procedure for measuring overall severity of psychiatric disturbance.. Arch Gen Psychiatry.

[77] Shi H, Vigneau-Callahan KE, Matson WR, Kristal BS (2002). Attention to relative response across sequential electrodes improves quantitation of coulometric array.. Anal Biochem.

[78] Schiavo S, Ebbel E, Sharma S, Matson W, Kristal BS (2008). Metabolite identification using a nanoelectrospray LC-EC-array integrated system.. Anal Chem.

[79] Eriksson L, Johansson E, Kettanah-Wold N, Wold S (2001). Multi- and megavariate data analysis..

[80] Hatzimanikatis V, Li C, Ionita JA, Broadbelt LJ (2004). Metabolic networks: enzyme function and metabolite structure.. Current Opinion in Structural Biology.

[81] Lehmann EL (1975). Nonparametrics: Statistical Methods Based on Ranks..

[82] Kendall M, Gibbons JD (1990). Rank Correlation Methods..

[83] Hastie T, Tibshirani R, Friedman J (2001). The Elements of Statistical Learning..

[84] Hastie T, Tibshirani R, Friedman J (2001). The Elements of Statistical Learning..

